# *Trichoderma* Counteracts the Challenge of *Phytophthora nicotianae* Infections on Tomato by Modulating Plant Defense Mechanisms and the Expression of Crinkler, Necrosis-Inducing *Phytophthora* Protein 1, and Cellulose-Binding Elicitor Lectin Pathogenic Effectors

**DOI:** 10.3389/fpls.2020.583539

**Published:** 2020-11-04

**Authors:** Federico La Spada, Claudia Stracquadanio, Mario Riolo, Antonella Pane, Santa Olga Cacciola

**Affiliations:** ^1^Department of Agriculture, Food and Environment (Di3A), University of Catania, Catania, Italy; ^2^Department of Agriculture, University Mediterranea of Reggio Calabria, Reggio Calabria, Italy; ^3^Council for Agricultural Research and Agricultural Economy Analysis, Research Centre for Olive, Citrus and Tree Fruit-Rende CS (CREA-OFA), Rende, Italy

**Keywords:** gene expression, antagonism, *Trichoderma asperellum*, *Trichoderma atroviride*, biological control, root rot, crown rot

## Abstract

Decoding the mechanisms of plant defense against plant pathogens in a scenario where antagonistic activity and the plant growth-promoting effects of useful organisms intervene simultaneously is a new frontier of plant pathology. Here, we demonstrated that (i) two selected strains of *Trichoderma asperellum* and *Trichoderma atroviride* promoted tomato (*Solanum lycopersicum*) growth and reduced the severity of disease caused by the oomycete *Phytophthora nicotianae* and (ii) the genetic patterns of the components of the experimental model system tomato–*Trichoderma* spp.–*P. nicotianae* were differentially expressed. The beneficial effects in both the promotion of the growth of host plant and the biological control of the pathogen by two selected strains of different *Trichoderma* species were tested both *in planta* and *in vitro*. In both respects, *T. atroviride* demonstrated to be more effective than *T. asperellum*. Additionally, the simultaneous transcriptional reprogramming of several plant defense-related genes, pathogen effectors, and mycoparasitism-related genes in tomato, *P. nicotianae*, and *Trichoderma* spp., respectively, was evaluated during the three-component interaction. Results support the hypothesis that *Trichoderma* spp. elicit the expression of plant defense-related genes. As expected, a mycoparasitism-related gene was significantly up-regulated in *Trichoderma*-colonizing tomato plants infected by *P. nicotianae*. Finally, a marked up-regulation of the genes encoding two necrosis-inducing effectors was observed in *P. nicotianae* infecting tomato plants colonized by *Trichoderma*. In conclusion, this study is a contribution toward understanding the genetic pathways related with the ability of *Trichoderma* spp. to counteract the challenge of *P. nicotianae* infections on tomato. Additionally, the experiments revealed the beneficial effects in the tomato growth promotion of a new *T. atroviride* strain and its good antagonistic effectiveness in the biological control of root and crown rot incited by *P. nicotianae*, confirming that *Trichoderma* spp. can be a powerful tool in integrated pest management strategies of *Phytophthora* diseases of horticultural crops.

## Introduction

In the last years, the biological control agents of plant pathogens, also known as antagonists, have inspired several research projects and the development of new strategies in the management of plant diseases (Ghazanfar et al., 2018).

The use of antagonistic microorganisms in plant protection is a low-risk practice for human health; moreover, the combination of these organisms with reduced levels of fungicides promotes a degree of disease suppression similar to that achieved with treatment using fungicide at normal doses (Monte, 2001; Benítez et al., 2004; Gilardi et al., 2020).

Among plant pathogens, phytopathogenic soil-borne fungi and oomycetes stand out since they are a threat to plant productivity on a global scale for a broad range of crops (Erwin and Ribeiro, 1996; Benítez et al., 2004; Mammella et al., 2011; Cacciola and Gullino, 2019).

The majority of applications of antagonistic microorganisms in the control of soil-borne plant diseases caused by fungal and oomycete pathogens have been conducted by selected strains of *Trichoderma* species (Benítez et al., 2004; Woo et al., 2014; Guzmán-Guzmán et al., 2019). The high effectiveness of *Trichoderma* spp. as biological control agents is due to both their antagonistic activity (Benítez et al., 2004) and the efficiency of these organisms in promoting plant growth and defense mechanisms (Harman et al., 2004; Verma et al., 2007; Shoresh et al., 2010; Singh and Islam, 2010; Tucci et al., 2011). The antagonistic activity of *Trichoderma* spp. can be considered the final result of different mechanisms, direct and indirect, acting synergistically to achieve disease control (Howell, 2003; Benítez et al., 2004). The indirect mechanisms include the competition for nutrients and space and the ability to produce metabolites that either inhibit spore germination, kill the cells (antibiosis), or modify the pH of rhizosphere. The direct interaction between antagonist and pathogen, usually indicated as mycoparasitism, includes both physical contact and the synthesis of hydrolytic enzymes, toxic compounds, and/or antibiotics that act synergistically to kill the pathogen (Benítez et al., 2004). Among hydrolytic enzymes, chitinases are the most relevant in mycoparasitism (Carsolio et al., 1994; Osorio-Hernández et al., 2016). High levels of these enzymes produced by *Trichoderma* spp. have been positively correlated with the inhibition in the growth of both fungi and oomycetes (Osorio-Hernández et al., 2016).

The colonization of the rhizosphere by *Trichoderma* spp. also produces direct positive effects on plants, promoting their growth and activating their defense mechanisms. It is well known that the interaction with microorganisms triggers two main defense mechanisms in plants that protect them against the infection (Shoresh et al., 2005). The first is known as systemic acquired resistance (SAR); this mechanism, which is considered to be triggered by local infection, can provide long-term resistance throughout the plant to subsequent infection by different pathogens. It is correlated with the synthesis of pathogenesis-related (PR) proteins, which is mediated by the up-regulation of genes encoding enzymes involved in the biosynthesis of salicylic acid (SA) (Zhang et al., 2010). The second mechanism, known as induced systemic resistance (ISR) and initially described in plants colonized by non-pathogenic rhizobacteria (Segarra et al., 2007), is correlated with the synthesis of jasmonic acid (JA) and ethylene (ET), which are mediated by the transcription factors MYC2 and ERF (Shoresh et al., 2005). This kind of resistance induces a primed state which enhances defense gene expression in the plant upon subsequent pathogen attack (Segarra et al., 2007). Since *Trichoderma* spp. are able to activate both kinds of resistances, the current hypothesis is that plants initially perceive their root colonization as a potential pathogen attack and then react with the activation of ISR mechanisms (Shoresh et al., 2005; Tucci et al., 2011).

Plant defensive mechanisms also comprise the synthesis of protective molecules acting directly against the pathogen; these include plant defensins, a family of small cationic peptides widely distributed among all plant families (Cui et al., 2018). These antimicrobial peptides proved to be very effective in the inhibition on the growth of pathogens (Lay and Anderson, 2005; Seo et al., 2014). Antifungal defensins have been identified in radish (Terras et al., 1992), pea (Mendenhall and Hodge, 1998; Almeida et al., 2000; Lobo et al., 2007; Gonçalves et al., 2012), and tomato (Cui et al., 2018). The sequencing of the whole genome of tomato (Sato et al., 2012) makes this plant a good model system for the study of plant-microorganisms interaction (Cui et al., 2018). With more than 75,000 ha, tomato is the most widely cultivated vegetable crop in Italy (ISTAT, 2020). Globally, among horticultural products, tomato ranks third for volumes of production–after potato and sweet potato–and first in terms of processing volumes (Brasesco et al., 2019). Tomato is susceptible to numerous diseases (Jones et al., 2016), among which root and crown rot incited by *Phytophthora* species represent one of the most important causes of yield losses (Erwin and Ribeiro, 1996; Jones et al., 2016). Several *Phytophthora* spp., including *Phytophthora capsici*, *Phytophthora cryptogea*, *Phytophthora drechsleri*, *Phytophthora infestans*, and *Phytophthora nicotianae*, have been reported to infect tomato worldwide (Pane et al., 2000; Jones et al., 2016). In Italy, *P. nicotianae* is the main species associated with the disease (Garibaldi and Gullino, 2010). *P. nicotianae* is worldwide recognized as one of the most devastating oomycete plant pathogens with a very broad host range of more than 255 plant species, including model plants such as *Nicotiana tabacum* and *Arabidopsis thaliana* (Kamoun et al., 2015; Panabières et al., 2016). The progress in the knowledge of the genomics of *P. nicotianae* makes it a suitable model to understand the molecular basis of pathogenesis of oomycete plant pathogens (Meng et al., 2014).

Plant pathogens secrete arsenals of proteins (effectors) that enable parasitic infection and reproduction (Birch et al., 2006; Stassen and Van den Ackerveken, 2011). Plants recognize the initial pathogen-associated molecular pattern (PAMPs) signals and activate pattern-triggered immunity (PTI) to counteract the further colonization by the pathogen. Successful pathogens have developed wide effector repertoires that not only function directly as toxins to induce plant cell death but can also suppress PTI and trigger susceptibility of the plant (Jones and Dangl, 2006). *Phytophthora* species also secrete a large array of effectors during infection of the plant hosts (Stam et al., 2013). Effectors of several species of *Phytophthora* have been identified (Chepsergon et al., 2020; McGowan et al., 2020). Among these, the *Crinkler* (CRN) proteins are a family of effectors that cause necrosis in the cells of the host and also induce further intracellular effectors that target the host nucleus during infection (Stam et al., 2013). The necrosis-inducing *Phytophthora* protein 1 (NPP1) is another important *Phytophthora* effector which has been associated with the induction of necrosis in parsley, *A. thaliana*, and potato (Fellbrich et al., 2002; Gijzen and Nürnberger, 2006). After artificial infiltration, this protein has also been observed to induce the transcription of PR-genes in *A. thaliana* leaves (Fellbrich et al., 2002). An additional important group of effectors includes cell wall glycoproteins named cellulose-binding elicitor lectin (CBEL); they have been found localized in the inner and outer layers of the *Phytophthora* mycelium cell walls and are present in close contact with the host cell during infection (Séjalon et al., 1995). Previous studies carried out on tobacco demonstrated that artificial infiltration with CBEL results in local necrosis of the infiltrated area and the induction of an array of defense responses (Séjalon-Delmas et al., 1997).

**FIGURE 1:**
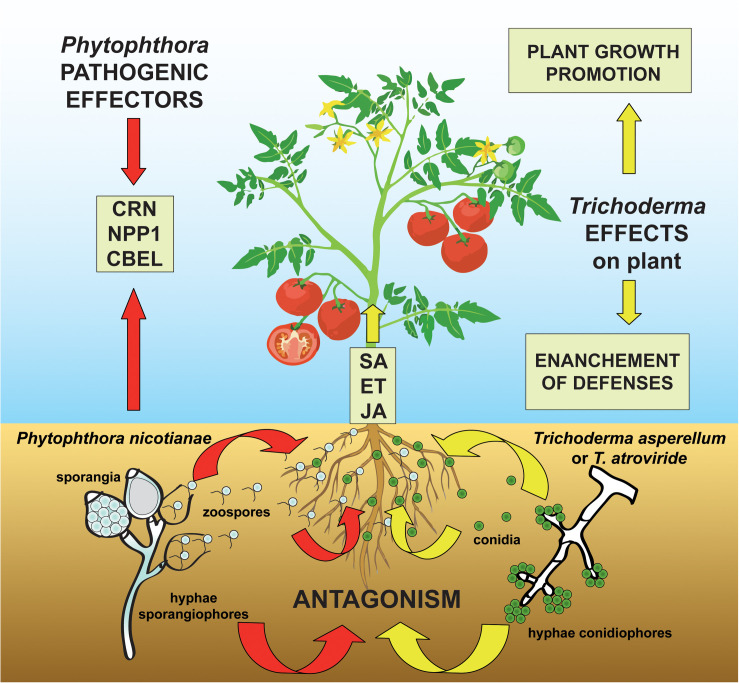
Proposed model for the three-way system plant-pathogen-antagonist showing how *Trichoderma* species can modulate the molecular signaling in the challenge between the oomycete pathogen *Phytophthora nicotianae* and the host plant tomato. The colonization of the tomato rhizosphere by *Trichoderma* spp. triggers both growth promotional effects and plant defense mechanisms by the elicitation of salicylic acid (SA)-, ethylene (ET)-, and jasmonic acid (JA)-dependent processes. At the same time, the *P. nicotianae*-parasitic infection process is mediated by the secretion of pathogenic effectors (including *Crinkler*–CRN–proteins, the necrosis-inducing *Phytophthora* protein 1–NPP1, and cellulose-binding elicitor lectin–CBEL–glycoproteins) which act to suppress the ET- and JA-plant response and whose synthesis is modified by the antagonistic interaction with *Trichoderma* spp.

As a consequence of the restrictions in the use of synthetic fungicides due to their toxicity to humans and animals as well as to their environmental impact, there has been growing interest in alternative approaches to chemical control of *P. nicotianae*, including the application of biocontrol agents (Singh and Islam, 2010; Gilardi et al., 2014a, b). Although the genomics of the infection process of host plants by *Phytophthora* spp. as well as the plant root colonization process and the mycoparasitism of fungal pathogens by *Trichoderma* spp. has been extensively investigated (Kubicek et al., 2011; Atanasova et al., 2013; Meng et al., 2014; Crutcher et al., 2015; Guzmán-Guzmán et al., 2017; Köhl et al., 2019; Morán-Diez et al., 2019; Ramírez-Valdespino et al., 2019; Chepsergon et al., 2020; Pachauri et al., 2020; Wang et al., 2020), there is limited information on the complex interaction of plant-beneficial antagonistic microorganism-pathogen, as analyzed in a comprehensive experimental workflow considering together physiological effects, metabolic pathways, and genes involved in this tripartite interaction (Figure 1).

To gain a better understanding of the events involved in the plant-microorganisms interaction, this study focuses on (i) the evaluation of the ability of two selected strains of *Trichoderma asperellum* and *Trichoderma atroviride* to promote the growth of *Solanum lycopersicum* and control root and crown rot incited by *P. nicotianae* and (ii) the identification of the main differentially expressed genes and metabolic pathways activated as a consequence of tripartite interaction in the experimental system tomato–*Trichoderma* spp.–*P. nicotianae*.

## Materials and Methods

### Fungal Isolates

The pathogen *P. nicotianae*, strain Ph_nic, was sourced from roots of a symptomatic plant of a local cultivar of *S. lycopersicum* in a nursery in Sicily. For the isolation, infected roots were firstly separated from the stem of the plant and washed with distilled water; then, washed roots were blotted dry and plated on selective PARPNH V8-agar (Jung et al., 2019) and examined under a stereomicroscope for the presence of *Phytophthora*-like coenocytic hyphae after 48 h of incubation at 28°C in the dark. Then, pieces cut from the advancing margins of the colony were sub-cultured on V8-agar Petri dishes and incubated at 28°C in the dark for a week. Purified cultures were finally obtained by single hyphal culture on V8-agar.

The antagonist *T. asperellum* strain IMI393899 (Puglisi et al., 2012; Cacciola et al., 2015), previously identified as *Trichoderma harzianum*, belonged to the collection of the Molecular Plant Pathology Laboratory (MPPL); Department of Agriculture, Food, and Environment (Di3A); University of Catania, while the *T. atroviride*, isolate TS, was obtained from the parasitized basidiocarp of a specimen of *Ganoderma lucidum* collected in Apulia (southern Italy). For the isolation of *T. atroviride*, infected tissues from the parasitized basidiocarp of *G. lucidum* were excised in 5-mm fragments, disinfected with 1% NaClO for 2 min, rinsed in sterile distilled water, and plated on Potato Dextrose Agar (PDA) amended with streptomycin sulfate at the concentration of 0.25 g/l. After 24 h of incubation at 25°C in the dark, growing colonies were sub-cultured on PDA plates. Purified cultures were finally obtained by single spore culture on PDA medium.

### Molecular Identification of Fungal and Oomycete Isolates

The identification of the isolates of *P. nicotianae* and *T. atroviride* was carried out by the amplification and analysis of the internal transcribed spacer (ITS) regions of the ribosomal DNA (rDNA). In this study, the DNA was extracted by using PowerPlant^®^ Pro DNA Isolation Kit following the manufacturer’s instructions. The amplifications of the ITS regions of the rDNA of *Phytophthora* and *Trichoderma* isolates were performed by using the *Taq* DNA polymerase, recombinant (Invitrogen^TM^) with the universal primer pairs ITS-6 (5′-GAAGGTGAAGTCGTAA CAAGG-3′) (Cooke et al., 2000) and ITS-4 (5′-TCCTCCGCTT ATTGATATGC-3′) (White et al., 1990) and ITS-5 (5′-GGAAGT AAAAGTCGTAACAAGG-3′) (White et al., 1990) and ITS-4, respectively. The PCR amplifications were carried out in a 25-μl reaction mix containing PCR buffer (1×), dNTP mix (0.2 mM), MgCl2 (1.5 mM), forward and reverse primers (0.5 μM each), *Taq* DNA polymerase (1 U), and 100 ng of DNA. The thermo-cycler conditions were as follows: 94°C for 3 min; followed by 35 cycles of 94°C for 30 s, 55°C for 30 s, and 72°C for 30 s; and then 72°C for 10 min. Obtained amplicons were detected in 1% agarose gel and sequenced in both directions by an external service (Macrogen). Obtained sequences were analyzed by using FinchTV v.1.4.0^1^. For species identification, blast searches in GenBank^2^ were performed.

Two representative isolates, namely, Ph_nic (*P. nicotianae*) and TS (*T. atroviride*), were randomly selected for further experimentations. *T. asperellum* strain IMI393899 had been previously identified (Puglisi et al., 2012; Cacciola et al., 2015) with the same procedure used for *T. atroviride* and stored in the collection of MPPL, Di3A.

### Treatment of Tomato Plants With *Trichoderma* Strains

In order to investigate the plant growth-promoting effect and induction of resistance to the pathogen infection by the symbiotic interaction with *Trichoderma* spp., tomato plants were grown in association with the selected *Trichoderma* strains at the root system.

The *Trichoderma*-tomato interaction was established in accordance with the method described by Tucci et al. (2011). Seeds of *S. lycopersicum* cv. Cuor di bue (Vilmorin Italia S.R.L., Funo, Bologna, Italy) were sterilized in 2% NaClO for 20 min, rinsed in sterile distilled water, and incubated in a conidial suspension (10^6^ conidia/ml) of either IMI393899 or TS; control seeds were suspended in water. Both treated and control seeds were air dried for 24 h and then sown in sterilized universal soil (©Cifo Srl, Giorgio di Piano, Bologna, Italy) in 40-well polystyrene trays and maintained in a growth chamber at 23°C, 80% relative humidity, and a photoperiod of 16 h of light and 8 h of dark. After 22 days, seedlings were transplanted in 200-cm^3^ plastic pots in sterilized universal soil. The positive-root colonization by *Trichoderma* was verified by re-isolation on PDA from roots of additional tomato control plants; the identity of the purified cultures of the *Trichoderma* strains was confirmed by PCR amplification and sequencing of their ITS region. These tomato plants will be called “*Trichoderma*-treated plants” in the text.

### Growth Promotion of Tomato Plants

Twenty-two-day-old tomato seedlings were grown with either strain IMI393899 of *T. asperellum* or strain TS of *T. atroviride*, which colonized the root systems and were grown for 12 weeks in a growth chamber at 23°C, 80% relative humidity, and a photoperiod of 16 h of light and 8 h of dark. Untreated seedlings (controls) were grown in the same conditions. A normal weekly irrigation was also provided.

The experimental set-up consisted of three treatments with 10 repetitions each: (i) untreated tomato plants (controls), (ii) tomato plants grown with *T. asperellum* strain IMI393899, and (iii) tomato plants grown with *T. atroviride* strain TS.

The promotion of the plant growth of “*Trichoderma*-treated plants” was then evaluated as follows: (i) weekly stem growth rate (cm/week), (ii) seedling total length, (iii) fresh root weight, and (iv) length at the end of the test (i.e., 12 weeks after the transplanting). All data were analyzed by using one-way ANOVA followed by Tukey’s honestly significant difference (HSD) test as a *post hoc* test (R software). Differences at *P* ≤ 0.05 were considered significant.

At the end of the test, *T. asperellum* strain IMI393899 and *T. atroviride* strain TS were re-isolated from roots of tomato plants from the respective treatments and then sequenced.

### Biological Control of *Phytophthora nicotianae*

#### *In vitro* Antagonistic Ability

The selected strains of *T. asperellum* and *T. atroviride* were screened for their ability to inhibit the mycelial growth (%) of *P. nicotianae* strain Ph_nic by *in vitro* dual culture assays. The formula applied was as follows:

$$\mathrm{Inhibition}\mathrm{of}\mathrm{growth}\left(\%\right)=\left(\frac{X-Y}{X}\right)\cdot 100$$

where,

*X* = growth of pathogen alone without antagonist (control)

and

*Y* = growth of pathogen along with the antagonist.

The dual culture test was carried out in 90-mm Petri dishes with 20 ml of PDA by placing 5-mm diameter agar plugs of the pathogen and antagonists taken from the margin of 1-week-old colonies grown on V8-agar and PDA, respectively. The dual culture was set by placing the pathogen and the antagonist 4 cm apart from each other. Furthermore, since the daily radial growth rate of the *P. nicotianae* strain Ph_nic was significantly lower than that of *Trichoderma* isolates, *P. nicotianae* was plated 72 h before the *Trichoderma* sp. Single cultures of the pathogen were used as control. Plates were incubated at 28°C in the dark, and radial mycelial growth was measured when *P. nicotianae* mycelium of control cultures covered the whole Petri dishes (namely, 13 days after incubation). Overall, the experimental set-up consisted of the following three treatments (including controls) made of 10 replicates each: (i) *P. nicotianae* isolate Ph_nic, (ii) *P. nicotianae* isolate Ph_nic + *T. asperellum* strain IMI393899, and (iii) *P. nicotianae* isolate Ph_nic + *T. atroviride* strain TS.

Data from the inhibition of the growth (%) of *P. nicotianae* 13 days from the beginning of the trial were analyzed by using one-way ANOVA followed by Tukey’s HSD test as a *post hoc* test (R software). Differences at *P* ≤ 0.05 were considered significant.

### *In planta* Antagonistic Ability

The *in planta* antagonistic ability of *T. asperellum* strain IMI393899 and *T. atroviride* strain TS toward *P. nicotianae*, strain Ph_nic, was demonstrated on tomato plants in a soil infestation test. Inoculum consisted of 12-day-old culture of the isolate Ph_nic grown at 25°C in a 750-ml jar containing 140 ml autoclaved V8-juice broth (200 ml/l juice and 800 ml/l distilled water amended with 3 g/l CaCO3) (Jung et al., 1996) and 170 ml of millet seeds. This experimental trial was carried out in two different steps which included a double treatment with the *Trichoderma* strains.

#### First Step

For the test, 4-month-old potted plants of *S. lycopersicum* cv. Cuor di bue, grown in sterilized universal soil with either *T. asperellum* strain IMI393899 or *T. atroviride* strain TS, in association with the root system (“*Trichoderma*-treated tomato seedlings”), were transplanted into 1,000-cm^3^ pots filled with a mixture of sterilized soil and the inoculum of Ph_nic prepared as described above (20 cm^2^ of inoculum per 1,000 cm^3^ of potting mixture). Untreated plants (controls) were not grown in association with *Trichoderma* strains; they were transplanted into pots filled with a mixture of sterilized soil and non-infested millet seed/V8-juice medium at the same rate.

#### Second Step

After transplanting, additional 100 ml of a conidial suspension (10^6^ conidia/ml) from *T. asperellum* IMI393899 and *T. atroviride* TS were provided to each plant from respective *Trichoderma* pre-treatment. All plants were then irrigated and maintained in a growth chamber at 23°C, 80% relative humidity, and a photoperiodic lighting of 16 h of light and 8 h of dark; a normal irrigation was also provided twice per week.

Overall, the experimental assay consisted of the following six treatments: (i) untreated tomato plants transplanted in a non-infested potting mixture (NI-PM); (ii) untreated tomato plants inoculated with infested potting mixture (I-PM); (iii) tomato plants treated with *T. asperellum* strain IMI393899 and transplanted in NI-PM; (iv) tomato plants treated with *T. asperellum* strain IMI393899 and transplanted in I-PM; (v) tomato plants treated with *T. atroviride* strain TS and transplanted in NI-PM; and (vi) tomato plants treated with *T. atroviride* strain TS and transplanted in I-PM. Each treatment included 10 replicates. The test was considered completed when plants of treatment (ii) showed severe symptoms of decay (i.e., 15 days after inoculation).

Plant damage was assessed on the basis of three different parameters: (i) wilting severity, visually evaluated in accordance with the empirical scale reported by Engelbrecht et al. (2007); (ii) fresh root weight; and (iii) fresh root length; the last two parameters were determined by separating the root system from the rest of the plant. The empirical scale used to rate the severity of wilting included the following values: 1 = normal (not wilted)–no signs of wilting or drought stress; 2 = slightly wilted–slight leaf angle changes but no folding, rolling, or changes in leaf surface structure; 3 = wilted–strong leaf angle change or protrusion of veins on the leaf surface but no cell death; 4 = severely wilted–very strong change of leaf angle or protrusion of veins on the leaf surface with initial necrosis; 5 = nearly dead–most leaves necrotic, some young leaves still green near the midrib, and leaf angles mostly near 0; 6 = dead–all above-ground parts dead.

At the end of the test, *T. asperellum* strain IMI393899, *T. atroviride* strain TS, and *P. nicotianae* isolate Ph_nic were re-isolated from plants of respective treatments and their identity was confirmed by PCR amplification and sequencing of their ITS region.

Data were analyzed by using one-way ANOVA followed by Tukey’s HSD test as a *post hoc* test (R software). Differences at *P* ≤ 0.05 were considered significant.

### Gene Expression in the Three-Way System Tomato–*Trichoderma* spp.–*Phytophthora nicotianae* Assay

#### Fungal Isolates

*Trichoderma asperellum* strain IMI393899 and *T. atroviride* strain TS were cultured on PDA for 7 days at 25°C, while *P. nicotianae* isolate Ph_nic was cultured in Petri dishes on V8-agar for 1 week at 28°C in the dark.

#### Tomato Plants

Tomato seeds (*S. lycopersicum* cv. Cuor di bue–Vilmorin Italia S.R.L., Funo, Bologna, Italy) were sterilized in 2% NaClO for 20 min, rinsed in sterile distilled water, and sown in an alveolar tray containing sterile vermiculite soaked in a nutrient solution (NS) prepared in accordance with Guérin et al. (2014) and Lebreton et al. (2018) with the following modifications: fertilizer 20-20-20 (Asso di Fiori-Cifo, S. Giorgio di Piano, Bologna, Italy) (0.1634 g/l), MgSO_4_ × 7H_2_O (0.15 g/l), FeNa-EDTA (40 mg/l). Trays were kept for 3 days in the dark at 23°C and 80% relative humidity; then, seedlings were transferred to a photoperiodic lighting (16 h of light:8 h of dark) and kept at the same temperature conditions and relative humidity for 30 days. Moreover, 30 ml of NS were provided once a week to renew the content of mineral salts; tomato plantlets were also watered twice a week. Seedlings were then transferred into plastic tubes containing 30 ml of NS.

#### *Trichoderma* spp. Colonization Assay

Thirty-day-old tomato seedlings growing in the aforementioned plastic tubes were treated with 300 μl of a suspension of germinated conidia (100 conidia/ml) of *T. asperellum* strain IMI393899 and *T. atroviride* strain TS. The suspension of germinated conidia of *Trichoderma* spp. was prepared as reported in Yedidia et al. (1999) with the following modifications: two flasks containing 100 ml each of a synthetic medium consisting of the aforementioned NS amended with 15 g/l of sucrose were autoclaved and then inoculated with 1 ml of conidial suspension (10^6^ conidia/ml) of each *Trichoderma* obtained from 7-day-old cultures grown on PDA medium; flasks were then shaken at 150 rpm for 24 h at 25°C to allow spore germination; after 24 h, tubes containing tomato seedlings were inoculated with 300 μl of the suspension of germinated conidia. Controls were inoculated with the NS amended with 15 g/l of sucrose.

#### *Phytophthora nicotianae* Infection Assay

Forty-eight hours after the treatment with *Trichoderma* spp., tomato seedlings were inoculated with zoospores of *P. nicotianae* (concentration: 100 zoospores/ml). *P. nicotianae* inoculum was prepared as follows: mycelial plugs from a 7-day-old culture of *P. nicotianae* grown on V8-agar were flooded with 20 ml of sterile distilled water and incubated at 25°C for 48 h under a constant fluorescent light. Zoospores were released in sterile distilled water by mature sporangia by placing mycelial plugs at 6°C for 1 h followed by another hour at 25°C. Zoospore concentration was measured by using a hemocytometer. Controls were inoculated with sterile distilled water.

#### Experimental Assay

Overall, the experimental assay consisted of the following six treatments: (i) untreated tomato seedlings; (ii) untreated tomato seedlings inoculated with *P. nicotianae* isolate Ph_nic; (iii) “*Trichoderma*-treated tomato seedlings” with *T. asperellum* strain IMI393899; (iv) “*Trichoderma*-treated tomato seedlings” with *T. atroviride* strain TS; (v) “*Trichoderma*-treated tomato seedlings” with *T. asperellum* strain IMI393899 and inoculated with *P. nicotianae* Ph_nic; and (vi) “*Trichoderma*-treated tomato seedlings” with *T. atroviride* strain TS and inoculated with *P. nicotianae* isolate Ph_nic. Each treatment was made up of six replicates. The test was considered completed (7 days after the inoculation of *P. nicotianae*) when seedlings of treatment (ii) showed severe symptoms of disease.

At the end of the test, seedlings from each treatment were collected and immediately frozen in liquid nitrogen and stored at −80°C. At the end of the test, *T. asperellum* strain IMI393899, *T. atroviride* strain TS, and *P. nicotianae* isolate Ph_nic were re-isolated and then sequenced, from additional seedlings from respective treatments.

#### RNA Isolation From Colonized Tomato Seedlings and cDNA Synthesis

Total RNA was extracted by using RNeasy Plant Mini Kit (Qiagen, Hilden, Germany) from frozen stem and roots from tomato seedlings (100 mg) ground to a fine powder with liquid nitrogen, following the protocol of the manufacturer and treated with TURBO DNA-free^TM^ Kit. RNA concentration was then adjusted to 200 ng/μl, and its quality was verified by performing a denaturing RNA electrophoresis gel in TAE agarose (Masek et al., 2005). Reverse transcription was performed by using High-Capacity cDNA Reverse Transcription Kit (Applied Biosystems^TM^, Foster City, CA, United States) following the manufacturer’s instructions.

#### Selection of Genes and Development of Specific Primers

Several genes from tomato, *Trichoderma* spp., and *P. nicotianae* involved in the tripartite interaction plant-antagonist-pathogen were selected (Supplementary Table S1). Both housekeeping and target genes from tomato and *P. nicotianae* were selected from previous studies (Tucci et al., 2011; Cui et al., 2018; Dalio et al., 2018). An NCBI nucleotide database^3^ search was carried out to select specific sequences from both endochitinase and housekeeping genes in *Trichoderma* spp. In order to obtain the highest primer specificity, sequences of genes LOC101262163, PR1b1, TomLoxA, SlyDF2, PpCRN4, PpCBEL4, PpNPP1.1, PpNPP1.3, PpNPP1.4, EF-1α, chi42, Gp_dh_N, and CHI18-5 (Supplementary Table S1) were directly derived from the respective genomic region as reported in the GenBank “whole genome shotgun sequencing project” of the respective organism (GenBank accession numbers: AEKE00000000.3–Tomato; MBGH00000000.1–*T. asperellum*; ABDG00000000.2–*T. atroviride*; AVGE00000000.1–*P. nicotianae*), and respective primer pairs were designed by using the Primer BLAST NCBI tool^4^; specificity of all selected primers was tested both by *in silico* (by using the Primer BLAST NCBI tool) and PCR amplification and sequencing of the target region.

#### Quantitative Real-Time PCR Analysis of Gene Expression

Amplifications were performed by using the iCycler iQ^TM^ Real-Time PCR Detection System (Bio-Rad). Reactions were performed in a total volume of 20 μl by mixing 10 ng of cDNA with 1 μl of 10 μM of each primer and 10 μl of PowerUp^TM^ SYBR^TM^ Green Master Mix (2×) (Applied Biosystems). Quantitative real-time PCR (qRT-PCR) experiments were carried out in triplicate. The thermo-cycling conditions were 2 min at 50°C (UDG activation) and 2 min at 95°C (Dual-Lock^TM^ DNA polymerase) followed by 40 cycles of two steps: 95°C for 15 s (denaturation) and 59°C or 60°C (annealing/extension) for 1 min. The quantification of gene expression was carried out by using the 2^–ΔΔ*Ct*^ method (Livak and Schmittgen, 2001). For each organism involved in the experiments, calibrator samples were represented by six replicates of the following: (i) untreated tomato seedling control samples and 7-day-old cultures of (ii) *P. nicotianae* isolate Ph_nic, (iii) *T. asperellum* strain IMI393899, and (iv) *T. atroviride* strain TS grown on NS-agarized medium (16 g/l of agar) amended with 15 g/l of sucrose.

The PCR efficiency was checked by standard curve Ct values vs. log (cDNA dilution). Curves were constructed by serial 10-fold dilution of cDNA for each primer pair; linear equations, determination coefficients (*R*^2^), and reaction efficiencies are given in Supplementary Table S2.

Data on gene expression were analyzed by using one-way ANOVA followed by Dunnett’s multiple comparisons test by using R software. Differences at *P* ≤ 0.05 were considered significant.

## Results

### Growth Promotion Assay

**FIGURE 2:**
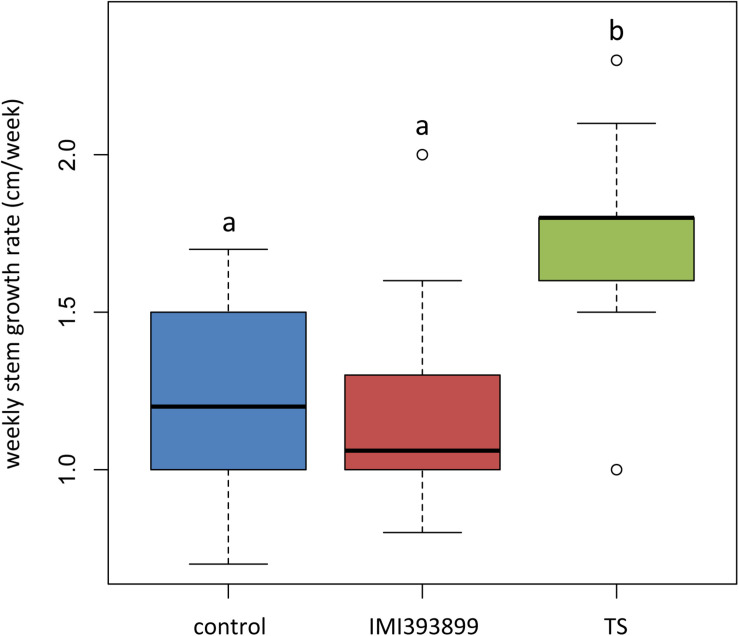
Effects of *Trichoderma asperellum* IMI393899 and *Trichoderma atroviride* TS treatments on the growth of *Solanum lycopersicum* cv. Cuor di bue seedlings. Weekly stem growth rate. Open and gray circles represent outliers and mean value data, respectively. Values sharing same letters are not statistically different according to Tukey’s honestly significant difference (HSD) test (*P* ≤ 0.05).

Results from growth promotion test showed that the growth of plants treated with *T. atroviride* strain TS was significantly stimulated compared with *T. asperellum* IMI393899-treated plants and untreated control plants (Figure 2). Overall, the weekly shoot growth of plants treated with *T. atroviride* strain TS was on average ca. 0.5 cm more than both the untreated and *T. asperellum*-treated plants, while the weekly shoot growth of *T. asperellum*-treated plants did not differ statistically from the untreated control (Figure 2).

**FIGURE 3:**
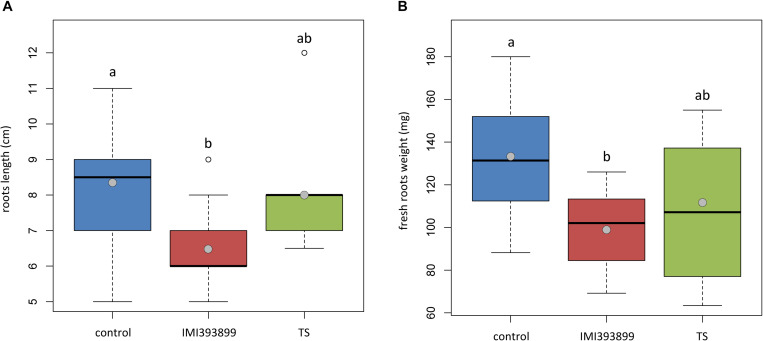
Effects of *Trichoderma asperellum* IMI393899 and *Trichoderma atroviride* TS treatments on the growth of *Solanum lycopersicum* cv. Cuor di bue seedlings. **(A)** Stem length and **(B)** fresh root weight at the end of the test. Open and gray circles represent outliers and mean value data, respectively. Values sharing same letters are not statistically different according to Tukey’s honestly significant difference (HSD) test (*P* ≤ 0.05).

**FIGURE 4:**
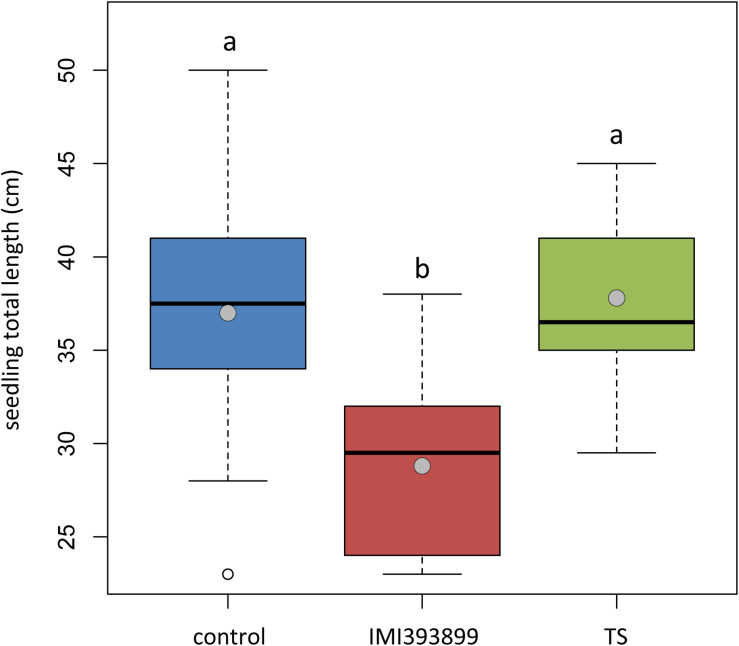
Effects of *Trichoderma asperellum* IMI393899 and *Trichoderma atroviride* TS treatments on the growth of *Solanum lycopersicum* cv. Cuor di bue seedlings. Seedling total length at the end of the test. Open and gray circles represent outliers and mean value data, respectively. Values sharing same letters are not statistically different according to Tukey’s honestly significant difference (HSD) test (*P* ≤ 0.05).

The same attitude in growth promotion was also confirmed by values of root length and fresh weight (Figures 3A,B). Overall, the treatment with *T. asperellum* IMI393899 reduced the growth of the root system of the plants, while plants treated with *T. atroviride* strain TS did not differ from the untreated control. The same trend was also observed for the total length of seedlings (Figure 4).

### Biological Control of *Phytophthora nicotianae*

#### *In vitro* Antagonistic Ability

Results from dual culture trial showed that both *Trichoderma*-tested isolates had an antagonistic effect on the growth of *P. nicotianae* (Table 1). In particular, the *T. atroviride* strain TS was more effective and inhibited the *P. nicotianae* growth by 63.50%, while *T. asperellum* strain IMI393899 inhibited the growth of *P. nicotianae* by 58.77% (differences between means were significant).

**TABLE 1:** *In vitro* antagonistic ability of *Trichoderma asperellum IMI393899* and *Trichoderma atroviride* test strains against *Phytophthora nicotianae* isolate Ph_nic.

Treatments	Mean mycelial radius of *P. nicotianae* (cm) ± standard deviations (SD)	Mean mycelial radius of *Trichoderma* sp. (cm) ± (SD)	Mean% of inhibition in growth ± (SD)
*Trichoderma asperellum* strain IMI393899	1.48 ± 0.10	2.50 ± 0.10	58.77 ± 2.80 a
*Trichoderma atroviride* strain TS	1.31 ± 0.08	2.69 ± 0,08	63.50 ± 1.93 b
*Phytophthora nicotianae* isolate Ph_nic	3.59 ± 0.00	–	–

*In the fourth column, values with different letters are statistically different according to Tukey’s honestly significant difference (HSD) test (*P* ≤ 0.05).*

#### *In planta* Antagonistic Ability

**FIGURE 5:**
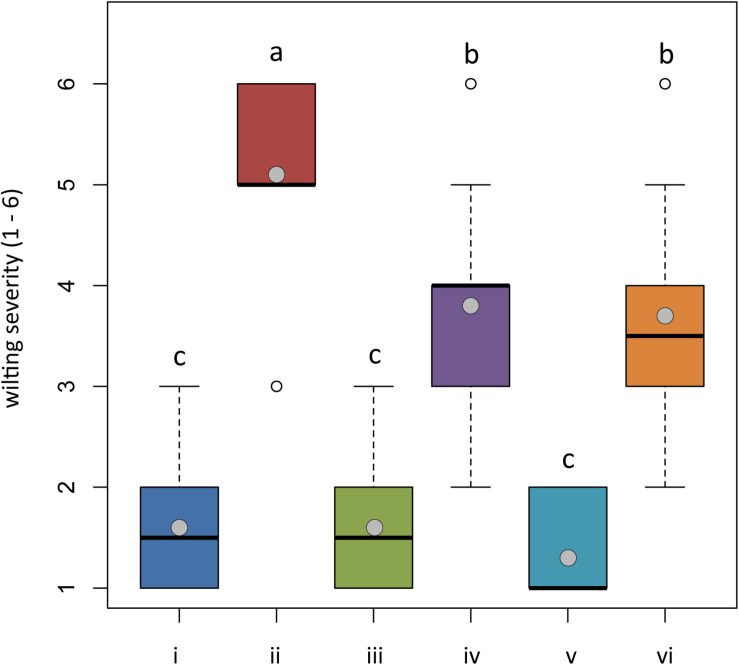
*In planta* antagonistic effectiveness of *Trichoderma* test strains against *Phytophthora nicotianae*. Wilting severity of 4-month-old *Solanum lycopersicum* cv. Cuor di bue developed from the following: (i) untreated tomato plants transplanted in a non-infested potting mixture (NI-PM); (ii) untreated tomato plants inoculated with infested potting mixture (I-PM); (iii) tomato plants treated with *Trichoderma asperellum* strain IMI393899 and transplanted in NI-PM; (iv) tomato plants treated with *T. asperellum* strain IMI393899 and transplanted in I-PM; (v) tomato plants treated with *Trichoderma atroviride* strain TS and transplanted in NI-PM; and (vi) tomato plants treated with *T. atroviride* strain TS and transplanted in I-PM. Open and gray circles represent outliers and mean value data, respectively. Values sharing same letters are not statistically different according to Tukey’s honestly significant difference (HSD) test (*P* ≤ 0.05).

**FIGURE 6:**
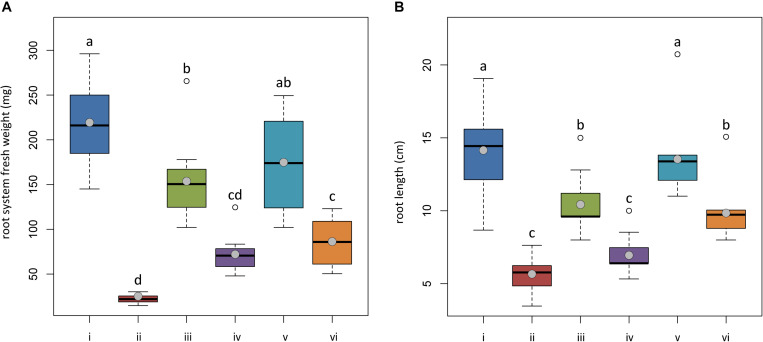
*In planta* antagonistic effectiveness of *Trichoderma* test strains against *Phytophthora nicotianae*. **(A)** Fresh root weight and **(B)** length of 4-month-old *Solanum lycopersicum* cv. Cuor di bue developed from the following: (i) untreated tomato plants transplanted in a non-infested potting mixture (NI-PM); (ii) untreated tomato plants inoculated with infested potting mixture (I-PM); (iii) tomato plants treated with *Trichoderma asperellum* strain IMI393899 and transplanted in NI-PM; (iv) tomato plants treated with *T. asperellum* strain IMI393899 and transplanted in I-PM; (v) tomato plants treated with *Trichoderma atroviride* strain TS and transplanted in NI-PM; (vi) tomato plants treated with *T. atroviride* strain TS and transplanted in I-PM. Open and gray circles represent outliers and mean value data, respectively. Values sharing same letters are not statistically different according to Tukey’s honestly significant difference (HSD) test (*P* ≤ 0.05).

At the end of the trial, both untreated plants and plants treated with *Trichoderma* spp. and transplanted into non-infested potting mixture (NI-PM), namely, those from treatments (i), (iii), and (v), were substantially asymptomatic showing a mean rating of wilting of 1.60, 1.60, and 1.30, respectively (Figure 5) (differences among means were not significant). They also showed a healthy root system, with the only exception of plants treated with *T. asperellum* IMI393899 that showed a statistically significant reduction of fresh weight and length compared with untreated controls (Figures 6A,B).

Plants inoculated with infested potting mixture (I-PM) showed severe symptoms of wilting (Figure 5) and a substantial reduction of root system (Figures 6A,B). However, the treatment with *T. atroviride* TS and *T. asperellum* IMI393899, with 40 and 60% of plant mortality, respectively, significantly reduced the mortality over untreated control plants (90% of mortality). Similarly, plants treated with *T. atroviride* TS and *T. asperellum* IMI393899 showed significant higher values of fresh root weight and length than untreated controls. However, overall, *T. atroviride* TS was more effective than *T. asperellum* IMI393899 in preventing root rot (Figures 6A,B).

### Gene Expression Levels in the Tripartite Interaction Tomato–*Trichoderma* spp.–*Phytophthora nicotianae*

#### Differences in the Expression of Tomato Defense-Related Genes

**FIGURE 7:**
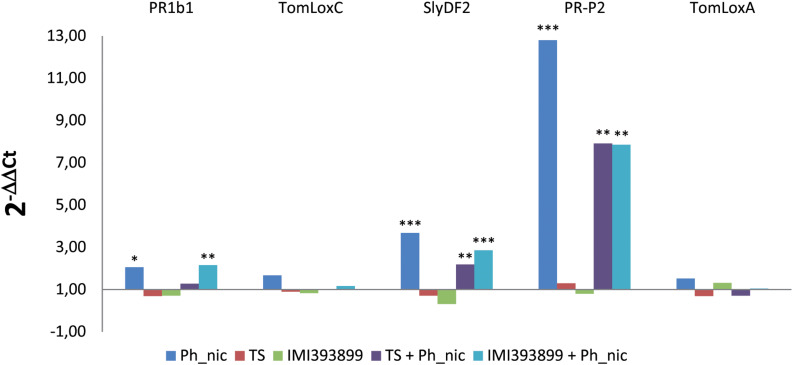
Differences in the expression levels of PR1b, TomLoxC, SlyDF2, PR-P2, and TomLoxA-encoding genes (GenBank accession numbers: Y08804.1, U37839.1, NM_001346524.1, X58548.1 and U09026.1, respectively) from 7-day-old *Trichoderma*-treated or non-treated *Solanum lycopersicum* cv. Cuor di bue seedlings inoculated or non-inoculated with *Phytophthora nicotianae*. Columns with asterisks are statistically different according to Dunnett’s test (^∗^*P* < 0.05, ^∗∗^*P* < 0.01, ^∗∗∗^*P* < 0.001), compared to their calibrator.

The defense mechanisms activated by tomato plants upon the simultaneous colonization of the root system by a root pathogen (*P. nicotianae*) and biocontrol agents (*Trichoderma* spp.) were evaluated on the basis of the expression of genes involved in the main plant defense pathways, namely, SA (i.e., PR proteins–*PR1b1* and *PR-P2*-encoding genes), JA (i.e., lipoxygenases enzymes–*TomLoxC* and *TomLoxA*-encoding genes), and a tomato plant defensin protein (i.e., *SlyDF2*-encoding gene) usually strongly involved in the tomato-*Phytophthora* sp. infection process (Cui et al., 2018; Figure 7).

Statistically significant reprogramming in the gene expression was observed for *PR1b1*, *PR-P2*, and *SlyDF2* in treatments that included the inoculation with the pathogen. PR1b1 was up-regulated in treatments (ii) (i.e., untreated tomato seedlings inoculated with *P. nicotianae* isolate Ph_nic) and (vi) (i.e., tomato seedlings treated with *T. atroviride* strain TS and inoculated with *P. nicotianae* isolate Ph_nic). *PR-P2* was strongly up-regulated in treatment (ii), while showed a lower up-regulation in treatments (v) (i.e., tomato seedlings treated with *T. asperellum* strain IMI393899 and inoculated with *P. nicotianae* Ph_nic) and (vi). Similarly, the tomato defensin *SlyDF2* was up-regulated only in treatments (ii), (v), and (vi). Both lipoxygenase-encoding genes (i.e., *TomLoxC* and *TomLoxA*) were expressed at similar levels in all treatments.

#### Differences in the Expression of *Phytophthora nicotianae* Pathogenic Effectors

**FIGURE 8:**
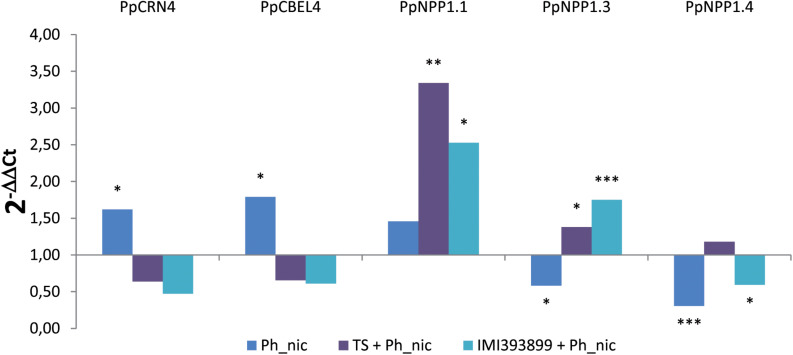
Differences in the expression levels of PpCRN4, PpCBEL4, PpNPP1.1, PpNPP1.3, and PpNPP1.4-encoding genes (GenBank accession numbers: ETM55095.1, ETM43740.1, ETM52620.1, ETM39327.1 and ETM36738.1, respectively) in *Phytophthora nicotianae* isolate Ph_nic from 7-day-old *Trichoderma*-treated or non-treated *Solanum lycopersicum* cv. Cuor di bue *P. nicotianae*-inoculated seedlings. Columns with asterisks are statistically different according to Dunnett’s test (^∗^*P* < 0.05, ^∗∗^*P* < 0.01, ^∗∗∗^*P* < 0.001), compared to their calibrator.

The effector expression of *P. nicotianae* was evaluated as differences in the relative expression levels of effector genes from different families: CRinkling and Necrosis effector PpCRN4; CBEL PpCBEL4; and three different members of the NEP1-like necrosis-inducing proteins PpNPP1.1, PpNPP1.3, and PpNPP1.4 (Figure 8).

Statistically significant differences were observed in the levels of all the effectors; both the *PpCRN4* and *PpCBEL4* genes were up-regulated only in untreated and inoculated seedlings [i.e., treatment (ii)], while were normally expressed on seedlings inoculated with the pathogen after being treated with TS of *T. atroviride* and IMI393899 of *T. asperellum* [i.e., treatments (v) and (vi), respectively]. Referring to the necrosis-inducing *Phytophthora* protein-encoding genes, *PpNPP1.1* showed a strong up-regulation in treatments (v), “*Trichoderma*-treated seedlings” with IMI393899 and inoculated with Ph_nic, and (vi), tomato seedlings treated with *T. atroviride* strain TS and inoculated with *P. nicotianae* isolate Ph_nic. On the contrary, *PpNPP1.3* was down-regulated in treatment (ii) and up-regulated in treatments (v) and (vi). Finally, *PpNPP1.4* was down-regulated in untreated seedlings and in treatment (v), “*Trichoderma*-treated seedlings” with IMI393899 and inoculated with Ph_nic.

#### Differences in the Expression of *Trichoderma* Antagonistic-Related Gene

**FIGURE 9:**
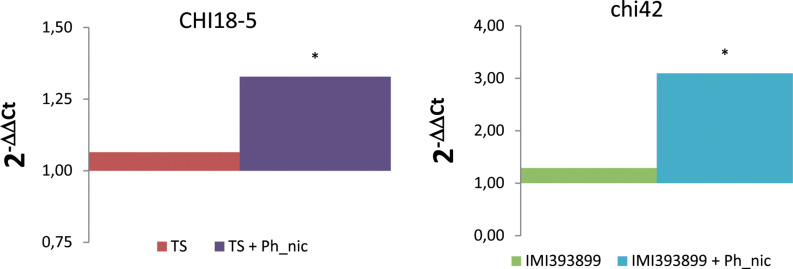
Differences in the expression levels of CHI18-5 (GenBank accession number: XM_014088210) and *chi42* (GenBank accession number: HM191684.1)-encoding genes, respectively, in *Trichoderma atroviride* strain TS (on the left) and *Trichoderma asperellum* strain IMI393899 (on the right) from 7-day-old *Phytophthora nicotianae*-inoculated or non-inoculated *Solanum lycopersicum* cv. Cuor di bue *Trichoderma*-treated seedlings. Columns with asterisks are statistically different according to Dunnett’s test (^∗^*P* < 0.05), compared to their calibrator.

The mycoparasitism of both *Trichoderma* test strains was assessed based on the differential expression of gene encoding for chitinases (i.e., *CHI18-5*-encoding gene for *T. atroviride*; *chi42*-encoding gene for *T. asperellum*). As expected, both *Trichoderma* strains up-regulated the respective selected endochitinase-encoding gene exclusively in the treatment with *Phytophthora*-inoculated seedlings (Figure 9).

## Discussion

This study provides a comprehensive assessment of physiological and molecular mechanisms involved in the complex three-way plant-antagonist-pathogen interaction. Firstly, the effectiveness in the promotion of growth of the model plant *S. lycopersicum* cv. Cuor di bue by two selected strains of *T. asperellum* and *T. atroviride* was compared. Previous studies revealed that different cultivated lines of tomato have a differential response in the promotion of growth by commercial strains of *Trichoderma* spp. (Tucci et al., 2011); the strains *T. atroviride* P1 and *T. harzianum* T22 induced statistically significant improvements in the development of the stem only in one and two (respectively) out of four different tomato lines (i.e., Corbarino, TA209, M82, and SM36), compared with untreated controls. A similar trend was also observed in the root weight with also a significant decrement for one variety (i.e., M82) treated with *T. harzianum* T22 (Tucci et al., 2011). In general, our results indicated that between the two *Trichoderma* species, only *T. atroviride* was significantly able to promote the weekly growth rate of the tomato cv. Cuor di bue. This result confirms the variability of the effects induced by *Trichoderma* spp. in the promotion of the growth of different tomato varieties. In order to acquire a complete evaluation of the antagonistic activity of the selected *T. asperellum* IMI393899 and *T. atroviride* TS strains against the *P. nicotianae* Ph_nic isolate virulent toward tomato seedlings, in this study, a dual culture test and an *in planta* antagonistic trial were carried out. Various *Trichoderma* spp., including *T. harzianum*, *Trichoderma viride*, *Trichoderma virens*, *T. asperellum*, *Trichoderma gamsii*, *Trichoderma longibrachiatum*, and *T. atroviride*, showed a good antagonistic activity both *in vitro* and *in vivo* against several soil-borne fungal pathogens (Smith, 1990; Al-mughrabi, 2008; Mastouri et al., 2010; Singh and Islam, 2010; Haggag and El-Gamal, 2012; Widmer, 2014; Yao et al., 2015; Ghazanfar et al., 2018). Results obtained here from *in vitro* tests show that both *Trichoderma* spp. tested strongly inhibited the growth of *P. nicotianae* isolate Ph_nic, with a more marked inhibition by *T. atroviride* TS over *T. asperellum* IMI393899. Similarly, the *in planta* antagonistic trial showed that *T. atroviride* TS was more effective than *T. asperellum* IMI393899 in the reduction of disease severity, even if none of them provided a complete control of the disease. These results are in agreement with those obtained in other similar pathogen-plant systems. Positive *in vitro* and *in planta* effectiveness by *Trichoderma* spp. was reported against virulent *Phytophthora* strains from pepper (Ezziyyani et al., 2017) and potato (Al-mughrabi, 2008). In accordance with previous studies, our results confirm that selected strains from *Trichoderma* spp. can represent a valid support in the integrated pest management strategies of *Phytophthora* diseases.

Decrypting the genetic pathways of plant defense mechanisms to counteract the plethora of effectors deployed by pathogens to develop the infection, concomitant with both the antagonistic activity and the growth-promoting effects of plant beneficial organisms, nowadays represents one of the main topics of modern plant pathology. Many studies have already investigated the modulation of the expression of protective molecules, pathogenic metabolites, and mucolytic enzymes in plants, pathogens, and antagonists, respectively, during a simplified two-way interaction (i.e., plant-antagonist, plant-pathogen, and antagonist-pathogen) (Carsolio et al., 1994; Tucci et al., 2011; Dalio et al., 2018). However, this compartmentalized approach does not clarify how the real mutual and simultaneous three-way interaction between the main actors involved in the biological control can reprogram their respective metabolic responses. In the present study, it has been investigated how the mutual gene-induced metabolic response of *S. lycopersicum* cv. Cuor di bue, *P. nicotianae*, and *Trichoderma* spp. is modified under the influence of the infection by the pathogen as well as of both the mycoparasitic and plant-beneficial activity of the antagonistic beneficial microorganism. To this aim, the modulation of the genetic pathways related with SA-dependent SAR (PR1b1 and PR-P2), ISR (TomLoxC and TomLoxA), and antifungal defensins (SlyDF2)-encoding genes were evaluated in tomato plants under *P. nicotianae* infection and the simultaneous *Trichoderma* spp. root colonization. The expression levels were compared with both *Trichoderma*-untreated–*P. nicotianae*-inoculated and *Trichoderma*-treated–*P. nicotianae*-non-inoculated tomato plants.

Among (SA)-dependent SAR and ISR-related genes, a statistically significant increment of transcripts was observed only in *PR1b1* and *PR-P2* transcript levels, while both analyzed *TomLoxC* and *TomLoxA* were normally expressed in all treatments. Previous studies have already demonstrated a significant variability between different *Trichoderma*-treated tomato varieties, including a widespread normal expression, in the levels of PR and ISR-related genes (Tucci et al., 2011). Considering also that root colonization by *Trichoderma* spp. activates only transiently the expression of defense-related genes (Yedidia et al., 1999, 2003; Shoresh et al., 2005; Segarra et al., 2009), results obtained here contribute to support the hypothesis that the colonization of roots by *Trichoderma* spp. could markedly take place only during the first phases of the interaction and then run out after a short time. Interestingly, the PR protein-encoding gene PR-P2 was activated more strongly in *Phytophthora*-inoculated and *Trichoderma*-untreated plants over treated and inoculated ones. The *PR-P2* is a PR4-encoding gene induced both by SA and wounding (Tornero et al., 1997; Bertini et al., 2003; Tucci et al., 2011). The expression of PR protein-encoding genes in *Trichoderma*-treated and pathogen-inoculated plants seems to be a mechanism characterized by a high spectrum of responses (Shoresh et al., 2005). A decreasing trend in the expression of PR-encoding genes was reported from different lines of tomato after the inoculation with *Botrytis cinerea* (Tucci et al., 2011); at the same time, proteomic studies on levels of PR proteins in *Trichoderma*-treated plants reported increments in pepper under *P. capsici* infection (Ezziyyani et al., 2017), in cucumber and maize (inoculated with *Pythium ultimum* and *Colletotrichum graminicola*, respectively) (Harman et al., 2004), and decrements in bean under leaf infection by *B. cinerea* and *Rhizoctonia solani* (Marra et al., 2006). By comparing the results obtained here with previous studies, it could then be speculated that the promotion of plant defenses by *Trichoderma* spp. is a mechanism affected by a variability of factors that could depend both on plant species and pathogens.

Additional significant increments of transcripts were observed in the levels of the tomato defensin SlyDF2-encoding gene. In this study, the evaluated tomato defensin gene was up-regulated only in *Phytophthora*-inoculated plants, including both *Trichoderma*-treated and untreated ones, while it was slightly down-regulated in *Trichoderma*-treated plants which did not receive *Phytophthora* inoculum. Plant defensins play a crucial role in the resistance of plants to pathogens (Penninckx et al., 1996). Antifungal activities were reported for several plant defensins, including RsAFP1 and RsAFP2 from radish (Terras et al., 1992), MsDef1 and MtDef4 from *Medicago* spp. (Gao et al., 2000), NaD1 from tobacco (Lay et al., 2003a, b), and Psd1 from pea (Almeida et al., 2000). However, in the last few years, the research has been mainly focused on the study of the potentiality of transgenic up-regulating defensin plants in the protection from fungal infections (Breen et al., 2015), resulting in a significant lack of knowledge about the modulation in the induction of defensins *in planta* under the pathogen/antagonist interaction. Results obtained here from the two-way interactions *Trichoderma*-tomato and *Phytophthora*-tomato agree with previous studies which described the induction of plant defensins by *Trichoderma* spp. in *A. thaliana* (Poveda et al., 2019) and the modulation of plant defensins by *Phytophthora* infection in tomato plants (Cui et al., 2018), respectively. However, the expression levels of defensins in tomato plants under the simultaneous *Trichoderma* root colonization and *Phytophthora* infection were not previously reported. In this study, the generalized reduction in the expression levels of the SlyDF2-encoding gene in *Trichoderma*-treated and *Phytophthora*-inoculated tomato plants over the untreated and *Phytophthora*-inoculated ones make it possible to speculate that the presence of *Trichoderma* control agents could reduce the intensity of this particular plant response.

In this study, the modulation of the transcription levels of the *P. nicotianae* genes encoding the effectors PpCRN4, PpCBEL4, PpNPP1.1, PpNPP1.3, and PpNPP1.4 was evaluated in *Phytophthora*-inoculated and *Trichoderma*-treated tomato plants and compared with untreated controls. Even though the role of *Phytophthora* effectors in the plant-pathogen interaction was previously reported (Séjalon et al., 1995; Séjalon-Delmas et al., 1997; Fellbrich et al., 2002; Gijzen and Nürnberger, 2006; Stam et al., 2013), this is the first study where the modulation of *P. nicotianae* effectors in a three-way tomato–*Trichoderma* spp–*Phytophthora* system has been evaluated.

Interestingly, in this study, among NPP1-encoding genes, a marked up-regulation was observed in the levels of *PnNPP1.1* and *PnNPP1.3* of *P. nicotianae* from *Trichoderma*-treated tomato plants over the untreated controls. The *P. nicotianae* necrosis-inducing *Phytophthora* protein 1 (NPP1) has been associated with the induction of necrosis in parsley and *A. thaliana* (Fellbrich et al., 2002), and a significant up-regulation of NPP1-encoding genes was reported for *P. nicotianae* during the infection of *Citrus sunki* and *Poncirus trifoliata* (Dalio et al., 2018). By comparing the expression levels from *Trichoderma*-untreated and -treated tomato plants, it could be speculated, therefore, that the antagonistic activity of *Trichoderma* spp. toward *P. nicotianae* could hamper the infection process of the plant, resulting in an up-regulation of the transcripts in this particular genetic pathway, which, in principle, should weaken the plant defenses, making the invasion of the host possible.

In this study, the *PpCRN4* and *PpCBEL4* genes showed both a generalized down-regulation in *Trichoderma*-treated tomato plants over the untreated ones. *PpCRN4* is a gene encoding a clinker (CRN) protein, while *PpCBEL4* encodes a cell wall glycoprotein named CBEL (Dalio et al., 2018). Both groups of effectors (i.e., CRN and CBEL) induce necrosis in plant tissue (Séjalon-Delmas et al., 1997; Stam et al., 2013). Dalio et al. (2018) observed that 3 days after inoculation, the expression levels of the *P. nicotianae PpCRN4*-encoding gene manifested differences that depended on the host plant, as the gene down-regulated and normally expressed by the pathogen in *C. sunki* and *P. trifoliata*, respectively. In the same plant-pathogen systems, *PpCBEL4*-encoding gene was up-regulated both in *C. sunki* and *P. trifoliata*. As already observed by other authors (Woo et al., 2006), the different response in the expression level of the pathogenic effectors of *P. nicotianae* could be explained by the great variety of molecular weapons of *Trichoderma* spp. These mechanisms can be activated in different combinations or at different steps of the infection process; they can be also triggered depending on the pathogen they are confronting or the plant they are colonizing. In particular, the up-regulation of *PnNPP1.1* and *PnNPP1.3* pathogenic effectors could be a response of *P. nicotianae* to the induction of the plant resistance mechanisms by *Trichoderma* spp. An analogous pattern has been already demonstrated by Marra et al. (2006) who observed, using a proteomic approach, an over production of the superoxide dismutase by *B. cinerea* in the three-way system *B. cinerea*-bean-*Trichoderma*. Conversely, the down-regulation of *PpCRN4* and *PpCBEL4* in *P. nicotianae* in the presence of *Trichoderma* spp. could be due to the antagonistic interaction between the microorganisms as well as the result of the enhancement of specific plant defenses by *Trichoderma* spp., which, in turn, could trigger in tomato plants a response similar to that constitutively activated by *C. sunki* plants infected by *P. nicotianae* (Dalio et al., 2018), thus determining the down-regulation of the *PpCRN4* gene. However, these pathogen genetic responses deserve to be further investigated.

Finally, in accordance with other authors (Osorio-Hernández et al., 2016), in this study, the level of endochitinase from both *T. asperellum* and *T. atroviride* was higher in *Trichoderma*-treated and *Phytophthora*-inoculated plants over the non-inoculated ones.

## Conclusion

This study provides the basis for understanding the complex and often unpredictable genetic interactions in a tripartite system, plant/beneficial organism/pathogen, instead of two, plant/pathogen, as in most systems studied so far. The experimental approach, including the individual components of the system, the host plant tomato, the oomycete *P. nicotianae*, i.e., the challenging pathogen, and the beneficial fungus *Trichoderma*, in all possible two-way combinations and the comparison with the three-way combinations made it possible to confirm or verify genetic mechanisms involved in the host-pathogen, host-growth-promoting beneficial organism, and pathogen-antagonistic beneficial organism interactions. Moreover, a better insight on how reciprocal interactions are modulated in more complex systems has been obtained. In particular, in this tripartite system, it was observed the simultaneous transcriptional reprogramming of plant defense-related genes, pathogen effectors, and mycoparasitism-related genes. Results support the hypothesis that *Trichoderma* spp. elicit the expression of plant defense-related genes and, as expected, a mycoparasitism-related gene was significantly up-regulated in *Trichoderma*-colonizing tomato plants infected by *P. nicotianae*.

Moreover, for the first time, it was observed that the *Trichoderma* treatment of tomato plants induced a marked up-regulation of the *P. nicotianae* pathogenic effectors *PnNPP1.1* and *PnNPP1.3* and, at the same time, a slight down-regulation of *PpCRN4* and *PpCBEL4*.

The findings that both the two- and three-way interactions vary with different *Trichoderma* species and the selection of a *T. atroviride* strain showing both a direct antagonistic activity against *P. nicotianae* and a growth-promoting effect on tomato plants are other interesting achievements of this study that have practical implications in the development and design of sustainable disease management strategies based on the application of biocontrol agents.

## Data Availability Statement

The datasets presented in this study can be found in online repositories. The names of the repository/repositories and accession number(s) can be found in the article/ Supplementary Material.

## Author Contributions

SOC, FLS, and AP conceptualized the study, analyzed the results, and reviewed and edited the draft. FLS, CS, and MR did the investigation and formal analysis and performed the experiments. SOC and AP were responsible for funding acquisition and supervised the study. FLS wrote the original draft. All authors reviewed the manuscript.

## Conflict of Interest

The authors declare that the research was conducted in the absence of any commercial or financial relationships that could be construed as a potential conflict of interest.

## Footnotes

1 https://digitalw orldbiology.com/FinchTV

2 http://www.ncbi.nlm.nih.gov/BLAST/

3 https://www.ncbi.nlm.nih.gov/nucleotide/

4 https://www.ncbi.nlm.nih.gov/tools/primer-blast/
