# Supplementary material for: Trichoderma Counteracts the Challenge of Phytophthora nicotianae Infections on Tomato by Modulating Plant Defense Mechanisms and the Expression of Crinkler, Necrosis-Inducing Phytophthora Protein 1, and Cellulose-Binding Elicitor Lectin Pathogenic Effectors
Source: Front Plant Sci. 2020 Nov 4;11:583539. doi: 10.3389/fpls.2020.583539 (PMC7672019; doi:10.3389/fpls.2020.583539)
Supplement: Supplementary file 1 [file Data_Sheet_1.docx]

**Table S1.** Gene-specific primers used in quantitative reverse transcription real-time polymerase chain reaction (qRT-PCR).

| **Target organism** | **Target gene (gene name)** | | **Ref.** | **GenBank accession number** | **Genebank genomic scaffold; respective base pair interval** | **Gene description** | **Primers** | **Melting temperature (°C)** | **Amplicone size (bp)** |
| --- | --- | --- | --- | --- | --- | --- | --- | --- | --- |
| *Lycopersicon esculentum* var. Cuor di bue | LOC101262163 | | (Tucci et al., 2011) | NM_001321306 .1 | NC_015448.3;  264146 - 264760 | actin-7-like  (housekeeping gene) | **Lesc_actin_For**  5'-AGTCAAGAGCCACATAGGCAAG-3' | 60.36 | 136 |
|  |  |  |  |  |  |  | **Lesc_actin_Rev**  5'-TTGGATCTTGCTGGTCGTGATTTA-3 | 60.56 |  |
|  |  | |  |  |  |  |  |  |  |
| - | PR1b1 | | (Tucci et al., 2011) | Y08804.1 | NW_020444107.1;  1578 - 2384 | ethylene-induced protein P1 | **Lesc_PR1b1-For**  5'-TTCATATTAGCAACATCAAAAGGGA-3' | 57.05 | 134 |
|  |  |  |  |  |  |  | **Lesc_PR1b1-Rev**  5'-CATATGAGACGTCGAGAAGTTAAAA-3' | 57.26 |  |
|  |  | |  |  |  |  |  |  |  |
| - | PR-P2 | | (Tucci et al., 2011) | X58548.1 | - | pathogenesis-related protein P2 | **Lesc_PR-P2-For**  5'-GGAACAGGAACACAAGAAACAGTGA-3' | 61.60 | 104 |
|  |  |  |  |  |  |  | **Lesc_PR-P2-Rev**  5'-CCCAATCCATTAGTGTCCAATCG-3' | 59.53 |  |
|  |  | |  |  |  |  |  |  |  |
| - | TomLoxA | | (Tucci et al., 2011) | U09026.1 | NC_015445.3;  3514080 - 3514426 | Lipoxygenase | **Lesc_LOX1.1-For**  5'-AAGGCATGTAATTCAGGGAGGA-3' | 59.15 | 100 |
|  |  |  |  |  |  |  | **Lesc_LOX1.1-Rev**  5'-TACCGAGAGACGAGCGTTTTG-3' | 60.40 |  |
|  |  | |  |  |  |  |  |  |  |
| - | TomLoxC | | (Tucci et al., 2011) | U37839.1 | - | Lipoxygenase | **Lesc_TomloxC-For**  5'-TCCGGCAACACCGTTTACTC-3' | 60.60 | 102 |
|  |  |  |  |  |  |  | **Lesc_TomloxC-Rev**  5'-GTCAATGGCCGGAAAATGTG-3' | 58.01 |  |
|  |  | |  |  |  |  |  |  |  |
| - | SlyDF2 | | (Cui et al., 2018) | NM_001346524.1 | NC_015444.3;  2394577 - 2395661 | defensin-like protein | **Lesc_SlyDF2-For**  5'-CCGAGGCACAATTCTTATCGC-3' | 59.74 | 101 |
|  |  |  |  |  |  |  | **Lesc_SlyDF2-Rev**  5'-CACAGAAATGGGACCAACGAG-3' | 59.19 |  |
|  |  | |  |  |  |  |  |  |  |
|  |  | |  |  |  |  |  |  |  |
| *Phytophthora nicotianae* | PPTG_08273 | | (Yan and Liou, 2006) | CK859493.1 | - | ubiquitin-conjugating enzyme (housekeeping gene) | **Pnic_ubc_F1**  5'-CCACTTAGAGCACGCTAGGA-3' | 58.90 | 152 |
|  |  |  |  |  |  |  | **Pnic_ubc_R1**  5'-TACCGACTGTCCTTCGTTCA-3' | 58.39 |  |
|  |  | |  |  |  |  |  |  |  |
|  |  | |  |  |  |  |  |  |  |
|  |  | |  |  |  |  |  |  |  |
|  |  | |  |  |  |  |  |  |  |
|  |  | |  |  |  |  |  |  |  |
|  | | *Cont. Table S1* | | | | | | | |
| **Target organism** | **Target gene (gene name)** | | **Ref.** | **GenBank accession number** | **Genebank genomic scaffold; respective base pair interval** | **Gene description** | **Primers** | **Melting temperature (°C)** | **Amplicone size (bp)** |
| *Phytophthora nicotianae* | PpCRN4 | | (Dalio et al., 2018) | ETM55095.1 | KI690828.1;  2085 - 2604 | Crinkler, citoplasmatic effector | **Pn-CRN-4-For**  5'-ATCTCTTCATCCGGCAACACT-3' | 58.57 | 100 |
|  |  |  |  |  |  |  | **Pn-CRN-4-Rev**  5'-TGATTCTGGACGAGTACCTCAA-3' | 59.44 |  |
|  |  | |  |  |  |  |  |  |  |
| - | PpCBEL4 | | (Dalio et al., 2018) | ETM43740.1 | KI693559.1;  85834 - 87327 | Cellulose-Binding, Elicitor, and Lectin activity, apoplastic effector | **PnCBEL-4-For**  5'-CGAGCAAACTAGCCTCCTATCT-3' | 59.37 | 111 |
|  |  |  |  |  |  |  | **PnCBEL-4-Rev**  5'-TGGAGGAAAAACCACGAGTGA-3' | 59.51 |  |
|  |  | |  |  |  |  |  |  |  |
| - | PpNPP1.1 | | (Dalio et al., 2018) | ETM52620.1 | KI691591.1;  35438 - 37514 | Necrosis-inducing Phytophthora Protein | **PnNPP1.1-For**  5'-ATCGGGTAGCGGCAACATAA-3' | 59.53 | 159 |
|  |  |  |  |  |  |  | **PnNPP1.1-Rev**  5'-GAAAACTCCGTTCCGTGTGG-3' | 59.42 |  |
|  |  | |  |  |  |  |  |  |  |
| - | PpNPP1.3 | | (Dalio et al., 2018) | ETM39327.1 | KI694660.1;  12734 - 13594 | Necrosis-inducing Phytophthora Protein | **PnNPP1.2-For**  5'-CAACGATGAACCTTGGTGCTG-3' | 60.07 | 125 |
|  |  |  |  |  |  |  | **PnNPP1.3-Rev**  5'-TTGCACGGTAGTGTTGGGAG-3' | 60.25 |  |
|  |  | |  |  |  |  |  |  |  |
| - | PpNPP1.4 | | (Dalio et al., 2018) | ETM36738.1 | KI695249.1  261 - 1010 | Necrosis-inducing Phytophthora Protein | **PnNPP1.4-For**  5'-TTGAGAACCCAAAGGTGAGCC-3' | 60.48 | 178 |
|  |  |  |  |  |  |  | **PnNPP1.4-Rev**  5'-CCAACAAGTCCCTAACACCGA-3' | 59.93 |  |
|  |  | |  |  |  |  |  |  |  |
|  |  | |  |  |  |  |  |  |  |
|  |  | |  |  |  |  |  |  |  |
| *Trichoderma asperellum* | EF-1α | |  | XM_024901686.1 | NW_020208831.1;  624759 - 624948 | elongation factor 1- alpha (housekeeping gene) | **Tasp_forEFTr**  5'-GAGGGACCCCAGAACATCAG -3' | 59.46 | 190 |
|  |  |  |  |  |  |  | **Tasp_revEFTr**  5'- GCTTTCACCGACTACCCTCC -3' | 60.11 |  |
|  |  | |  |  |  |  |  |  |  |
| - | chi42 | |  | HM191684.1 | NW_020208843.1;  32839 - 34296 | Endochitinase | **Tasp-chi42-For**  5'-GAACTTCCAAGCAGACGGCA-3' | 60.88 | 122 |
|  |  |  |  |  |  |  | **Tasp-chi42-Rev**  5'-AGTGCTTCTGATAATCGGCGT-3' | 59.86 |  |
|  |  | |  |  |  |  |  |  |  |
|  |  | |  |  |  |  |  |  |  |
|  |  | |  |  |  |  |  |  |  |
|  |  | |  |  |  |  |  |  |  |
|  |  | |  |  |  |  |  |  |  |
|  | | *Cont. Table S1* | | | | | | | |
| **Target organism** | **Target gene (gene name)** | | **Ref.** | **GenBank accession number** | **Genebank genomic scaffold; respective base pair interval** | **Gene description** | **Primers** | **Melting temperature (°C)** | **Amplicone size (bp)** |
| *Trichoderma atroviride* | Gp_dh_N | |  | XM_014091690.1 | NW_014013639.1;  430830 - 432942 | Glyceraldehyde 3-phosphate dehydrogenase, (housekeeping gene) | **Tatr_Gp_dh_N-For**  5'-TTTGCACCCTGCTTTGTCTTT-3' | 59.17 | 119 |
|  |  |  |  |  |  |  | **Tatr_Gp_dh_N-Rev**  5'-AAGGTCGATGCCTCCAAGAAAT-3' | 60.03 |  |
|  |  | |  |  |  |  |  |  |  |
| - | CHI18-5 | |  | XM_014088210 | NW_014013632.1;  2753800 - 2755269 | Endochitinase | **Tatr-CHI18-5-For**  GATCTTGCTTGCGGGAACAC | 59.83 | 148 |
|  |  |  |  |  |  |  | **Tatr-CHI18-5-Rev**  GGCCTACGACTATGCTGGTT | 59.54 |  |

**Table S2.** Linear equations, determination coefficients (R^2^) and reaction efficiencies obtained by plotting cDNA concentrations (log ng) and Ct values experimentally achieved by real-time PCR for each gene evaluated in this study.

| **Target organism** | **Target gene (gene name)** | **Type of material (sample and/or calibrator)** | **Linear equation** | **R^2^** | **Reaction efficiency (%)** |
| --- | --- | --- | --- | --- | --- |
| *Lycopersicon esculentum* var. Cuor di bue | LOC101262163 | Sample and calibrator | y = -3.2992x + 22.507 | 0.998 | 100.96 |
| - | PR1b1 | Sample and calibrator | y = -3.3398x + 19.452 | 0.999 | 96.92 |
| - | PR-P2 | Sample and calibrator | y = -3.3483x + 21.975 | 0.9994 | 98.91 |
| - | TomLoxA | Sample and calibrator | y = -3.3013x + 23.841 | 0.9954 | 100.87 |
| - | TomLoxC | Sample and calibrator | y = -3.3031x + 25.529 | 0.9969 | 100.79 |
| - | SlyDF2 | Sample and calibrator | y = -3.326x + 28.497 | 0.9983 | 99.83 |
|  |  |  |  |  |  |
| *Phytophthora nicotianae* | PPTG_08273 | Sample | y = -3.3018x + 21.534 | 0.9979 | 100.85 |
| - | PPTG_08273 | Calibrator | y = -3.3011x + 21.492 | 0.9991 | 100.88 |
| - | PpCRN4 | Sample | y = -3.3239x + 24.39 | 0.9983 | 99.92 |
| - | PpCRN4 | Calibrator | y = -3.3221x + 24.38 | 0.9993 | 99.99 |
| - | PpCBEL4 | Sample | y = -3.3075x + 23.129 | 0.9992 | 100.61 |
| - | PpCBEL4 | Calibrator | y = -3.3192x + 23.104 | 0.9995 | 100.11 |
| - | PpNPP1.1 | Sample | y = -3.4009x + 22.899 | 0.9984 | 96.81 |
| - | PpNPP1.1 | Calibrator | y = -3.3995x + 22.937 | 0.9987 | 96.86 |
| - | PpNPP1.3 | Sample | y = -3.3154x + 21.383 | 0.9934 | 100.27 |
| - | PpNPP1.3 | Calibrator | y = -3.3025x + 21.363 | 0.9997 | 100.82 |
| - | PpNPP1.4 | Sample | y = -3.3299x + 21.135 | 0.9998 | 99.67 |
| - | PpNPP1.4 | Calibrator | y = -3.326x + 21.157 | 0.9994 | 99.83 |
|  |  |  |  |  |  |
| *Trichoderma asperellum* | EF-1α | Sample | y = -3.2986x + 16.077 | 0.9995 | 100.98 |
| - | EF-1α | Calibrator | y = -3.2998x + 16.071 | 0.9996 | 100.93 |
| - | chi42 | Sample | y = -3.3121x + 21.971 | 0.9981 | 100.41 |
| - | chi42 | Calibrator | y = -3.3149x + 21.943 | 0.9966 | 100.29 |
|  |  |  |  |  |  |
| *Trichoderma atroviride* | Gp_dh_N | Sample | y = -3.343x + 19.58 | 0.9984 | 99.13 |
| - | Gp_dh_N | Calibrator | y = -3.3302x + 19.595 | 0.9994 | 99.66 |
| - | CHI18-5 | Sample | y = -3.3108x + 19.411 | 0.999 | 100.47 |
| - | CHI18-5 | Calibrator | y = -3.3051x + 19.419 | 0.9979 | 100.71 |
